# Activation of DNA Transposons and Evolution of piRNA Genes Through Interspecific Hybridization in *Xenopus* Frogs

**DOI:** 10.3389/fgene.2022.766424

**Published:** 2022-01-31

**Authors:** Kosuke Suda, Shun R. Hayashi, Kei Tamura, Nobuhiko Takamatsu, Michihiko Ito

**Affiliations:** Department of Bioscience, School of Science, Kitasato University, Sagamihara, Japan

**Keywords:** hybrid, transposable elements, piRNA cluster, allotetraploidization, reproductive isolation

## Abstract

Interspecific hybridization between two closely related species sometimes resulted in a new species with allotetraploid genomes. Many clawed frog species belonging to the *Xenopus* genus have diverged from the allotetraploid ancestor created by the hybridization of two closely related species with the predicted L and S genomes. There are species-specific repeated sequences including transposable elements in each genome of organisms that reproduce sexually. To understand what happened on and after the hybridization of the two distinct systems consisting of repeated sequences and their corresponding piRNAs, we isolated small RNAs from ovaries and testes of three *Xenopus* species consisting of allotetraploid *X. laevis* and *X. borealis* and diploid *X. tropicalis* as controls. After a comprehensive sequencing and selection of piRNAs, comparison of their sequences showed that most piRNA sequences were different between the ovaries and testes in all three species. We compared piRNA and genome sequences and specified gene clusters for piRNA expression in each genome. The synteny and homology analyses showed many distinct piRNA clusters among the three species and even between the two L and/or S subgenomes, indicating that most clusters of the two allotetraploid species changed after hybridization. Moreover, evolutionary analysis showed that DNA transposons including *Kolobok* superfamily might get activated just after hybridization and then gradually inactivated. These findings suggest that some DNA transposons and their piRNAs might greatly influence allotetraploid genome evolution after hybridization.

## Introduction

Interspecific hybridization, which is the crossing of two species, plays an important role in evolution. Many events of whole genome duplication (WGD) occurred during interspecific hybridization. For example, hybridization was believed to be involved in the two-rounds of WGD in the common ancestor of vertebrates (Ohno, 1970; Holland et al., 1994; Spring, 1997). Recently, Simakov et al. (2020) reported that the second WGD occurred in the mid–late Ordovician by interspecific hybridization. The allotetraploid frog *Xenopus laevis* diverged from the hybrid between two closely related diploid *Xenopus* species. Session et al. (2016) reported that the allotetraploid *X. laevis* origin should have had two distinct subgenomes with distinct families of transposable elements (TEs), and that the two diploid progenitor species diverged about 34 million years ago (mya) and hybridized approximately 17–18 mya. Hybridogenesis, the elimination of one of the parental genomes in the germline cells and gamete production of the other parental species is another interesting phenomenon in interspecific hybridization as observed in the edible frog *Pelophylax esculentus* (Miura et al., 2021).

We speculated that there are some significant differences of repeated sequences including transposons between two closely-related species, although the two genomes should have only a few differences of gene sequences. Transposons, also known as transposable elements (TEs), are mobile DNA sequences that can proliferate and are scattered in the genomes. Most multicellular organisms possess a large proportion of TEs in their genomes. Their selfish activity induces genome instability in various cells, including germ line cells (Belancio et al., 2008). In particular, genomic information in germ line cells should be properly inherited for subsequent generations. It has been known that PIWI-interacting RNAs (piRNA) belong to small noncoding RNAs and function in protection of genomic information from transposons in animal germ line cells. The complexes between piRNAs and PIWI are involved in epigenetic and post-transcriptional silencing of transposons against their expansion and invasion (Klattenhoff et al., 2007; Seto et al., 2007; Siomi et al., 2011). Precursor piRNAs are processed into mature piRNAs consisting of 25–32 nucleotides. Precursor piRNAs are transcribed from genomic regions termed piRNA clusters, which contain various types of transposons (Siomi et al., 2011). Transposons vary and evolve dramatically across species. Thus, piRNA-encoding piRNA clusters must have evolved concomitantly. However, little is known about the molecular coevolution of piRNA clusters and transposons. Moreover, the influence of interspecific hybridization on the transposon-piRNA system in vertebrates has not been reported, yet.

How did the mixture of the two different transposon-piRNA systems by interspecific hybridization influenced *Xenopus* allotetraploid genome evolution? To clarify the evolutionary overview of transposons and transposon-derived piRNAs in genomes after interspecific hybridization, we first identified piRNAs by small RNA-seq from female and male gonads of three *Xenopus* species consisting of hybrid-derived allotetraploid species, *X. laevis* and *X. borealis*, and one diploid species *X. tropicalis* as a non-hybrid control. An ancestral species of *X. tropicalis* diverged from the common one between *X. laevis* and *X. borealis* about 48 mya (Session et al., 2016). From the piRNA sequence and genome information, we found that piRNA clusters were not well conserved between the L and S subgenomes of *X. laevis*. Moreover, evolutionary analysis of transposons indicated that some DNA transposon superfamilies might become active just after hybridization. Finally, we discuss the relationships between “transposon-piRNA” system and interspecific hybridization.

## Material and Methods

### Databases

Genomic sequence information from *X. laevis* and *X. tropicalis* was obtained from Xenbase (www.xenbase.org/entry/), including *X. laevis* v9.2 and X. tropicalis v9.1 genome assemblies. Repbase (www.girinst.org/repbase/) was used for the transposon libraries of *X. laevis* and *X. tropicalis*. The *Xenopus* transposon library was constructed by merging the two libraries.

### Small RNA Extraction and Small RNA-Seq

Ovaries from adult female frogs were cut into small pieces, flash-frozen in liquid nitrogen, and stored. Testes from adult male frogs were flash-frozen in liquid nitrogen. Small RNAs were purified using a small RNA isolation kit (ISOGEN II, Nippon gene, Japan) according to the manufacturer’s protocol, except for the percentage change in ethanol from 75 to 90%. piRNAs were separated on a 15% polyacrylamide TBE-urea gel, and 20–50 base pair long were excised from the gel. They were then recovered using a small RNA PAGE recovery kit (Zymo Research, California, United States). Library construction and small RNA sequences were obtained from DNAFORM (Yokohama, Japan).

### Genome Assembly and Gene Annotation of *X. borealis*

Scaffolds were assembled using ABySS (github.com/bcgsc/abyss) under the conditions of (k = 83 B = 30G H = 3 kc = 3 v = −v] with *X. borealis* female or male whole genome sequences, SRR6357673 and SRR6357672, respectively. The resulting female and male scaffolds were integrated, and reference-guided scaffolding was performed with RaGOO (Alonge et al., 2019) -T sr using *X. laevis* v9.2 genome assembly as a reference. Gene annotation was carried out using BRAKER (Hoff et al., 2019). Protein sequence data were obtained from *Xenopus laevis* v2 (GCA_001663975.1_Xenopus_laevis_v2/), and the annotation data were filtered using gFACS (Caballero and Wegrzyn, 2019) at two statuses (monoexonic gene: min-exon-size 20 min-intron-size 20 min-CDS-size 900 —statistics-at-every-step—no-processing—rem-multiexonics—rem-genes-without-start-and-stop-codon—canonical-only, multiexonic gene: -min-exon-size 20 min-intron-size 20 min-CDS-size 300 —statistics-at-every-step—no-processing—rem-monoexonics—rem-genes-without-start-and-stop-codon—canonical-only). Gene protein sequences were extracted using SeqKit (bioinf.shenwei.me/seqkit/). Predicted protein sequences were aligned to those in *X. laevis* using BLASTP and then filtered using an E-value < 0.05.

### Classification of DNA Sequences in Genomes

In each genome, repeated sequences were mapped and classified using RepeatMasker as described above, and then exons, introns, and others were specified with BEDTools intersect.

### Identification of Transposable Elements in Genomes

The genomic transposon library from *X. borealis* was constructed using RepeatModeler (github.com/Dfam-consortium/TETools). The resulting *X. borealis* transposon library was incorporated into the transposon library, which was described above. The genomic locations of transposons were identified using RepeatMasker (www.repeatmasker.org/). Transposon diversity was recalculated using MAFFT (www.mafft.cbrc.jp/alignment/software/) —retree 2 —reorder and FastTree (www.microbesonline.org/fasttree/) -nt. A customized Python script was used for overlapping locations of transposons to give priority to low diversity scores. TE landscapes were basically generated by using RepeatLandscape (github.com/caballero/RepeatLandscape) to calculate Kimura distance as a measure of age.

### Identification of piRNAs and Their Clusters

Protein-coding RNAs and miRNAs were excluded from small RNA raw sequence reads using unitas that is out-of-the-box ready software for complete annotation of small RNA sequence datasets (Gebert et al., 2017), and 24 to 32 base reads were extracted using SeqKit (bioinf.shenwei.me/seqkit/). The resulting RNA reads were mapped to genome sequence data using Bowtie (bowtie-bio.sourceforge.net/index.shtm) −l.18 -n 0 -a—best—strata, and then treated with customized Python script, which allows alignments with two mismatches in the area except for the seed sequence and the last two sequences under the condition of sorted ELAND format in exchange of SAM format. In each read, one minimum mismatch alignment was selected with customized Python script. The counts of the resulting selected alignments mapped on the specific chromosome position were recalculated using reallocate (www.smallrnagroup.uni-mainz.de/software.html) 10000 1000b 1. Candidates of piRNA clusters were selected by using proTRAC (Rosenkranz and Zischler, 2012). The clusters were then identified by removing piRNAs that were lower than 1 loci/kb and 10 loci/piRNA clusters with awk and customized python scripts. Because proTRAC was useful for detecting a high density of piRNAs in a relatively wide region of the genome, we also used Stringtie (Pertea et al., 2015) for detecting the clusters in a relatively narrow region of the genome as performed by Toombs et al. (2017). In *X. tropicalis* and *X. laevis*, they were identified using customized Python script under the conditions of (Stringtie -m 50 -g 50 and -merge -m 500 -g 250 -F 50). In *X. borealis*, whose genome information has many 100 bp gaps, piRNA clusters were detected using customized Python script under the conditions of (Stringtie -m 150 -g 50 and -merge -m 500 -g 250 -F 50). In summary, piRNA clusters were determined by detection using proTRAC and/or Stringtie. piRNA clusters with more than half of the overlapped sequences in the specified region between female and male gonads were defined as the common ones.

### Analysis of 1 U/10 A and Overlapped Plus/Minus piRNA Strands

10 base DNA sequence corresponding to 5′ of each piRNA was extracted in the recalculated mapping ELAND format file which was described in the above paragraph, and changed to RNA sequence using seqkit seq–dna2ma. 1 U/10 A was plotted using WebLogo (weblogo.berkeley.edu).

For plus/minus strands of piRNAs, the ELAND format file was exchanged into BED format. Then, using awk script, each piRNA sequence was mapped in the genome and identified as plus or minus strand. Overlapped piRNA sequences were found using BEDTools intersect (Quinlan and Hall. 2010). The overlap score was measured as the product of plus and minus reads. The 10 nt Z-score was calculated from the mean and standard deviation of ratio of the overlap score on each length to total ones.

### Component of Genome and piRNA Clusters

Gene and exon loci were extracted for each *Xenopus* gene annotation and overlapped loci were merged using BEDTools merge. The classification of the genome and piRNA clusters were done in order of transposon, gene, exon, to avoid overlapping other components with BEDTools intersect.

### Evolutionary Analysis of piRNA Clusters

Synteny analysis of piRNA clusters with lengths longer than 1 kb was performed as follows. First, localizations of 10 exons up- and downstream of piRNA clusters were examined in the corresponding genome, and the genomic sequences were extracted. Neighboring BLAST hits were grouped into contiguous exons. Second, syntenic relationships between piRNA clusters were verified using more than three homologous exons and their bit scores. Third, we identified homologous sequences to piRNA clusters and their corresponding transposons in the query genome using blastn-task dc-megablast. BLAST hits below the relative size (10%) were discarded.

## Results

### A Variety of piRNA Molecules From Ovaries and Testes in the Three *Xenopus* Species, *X. tropicalis*, *X. leavis*, and *X. borealis*

**FIGURE 1:**
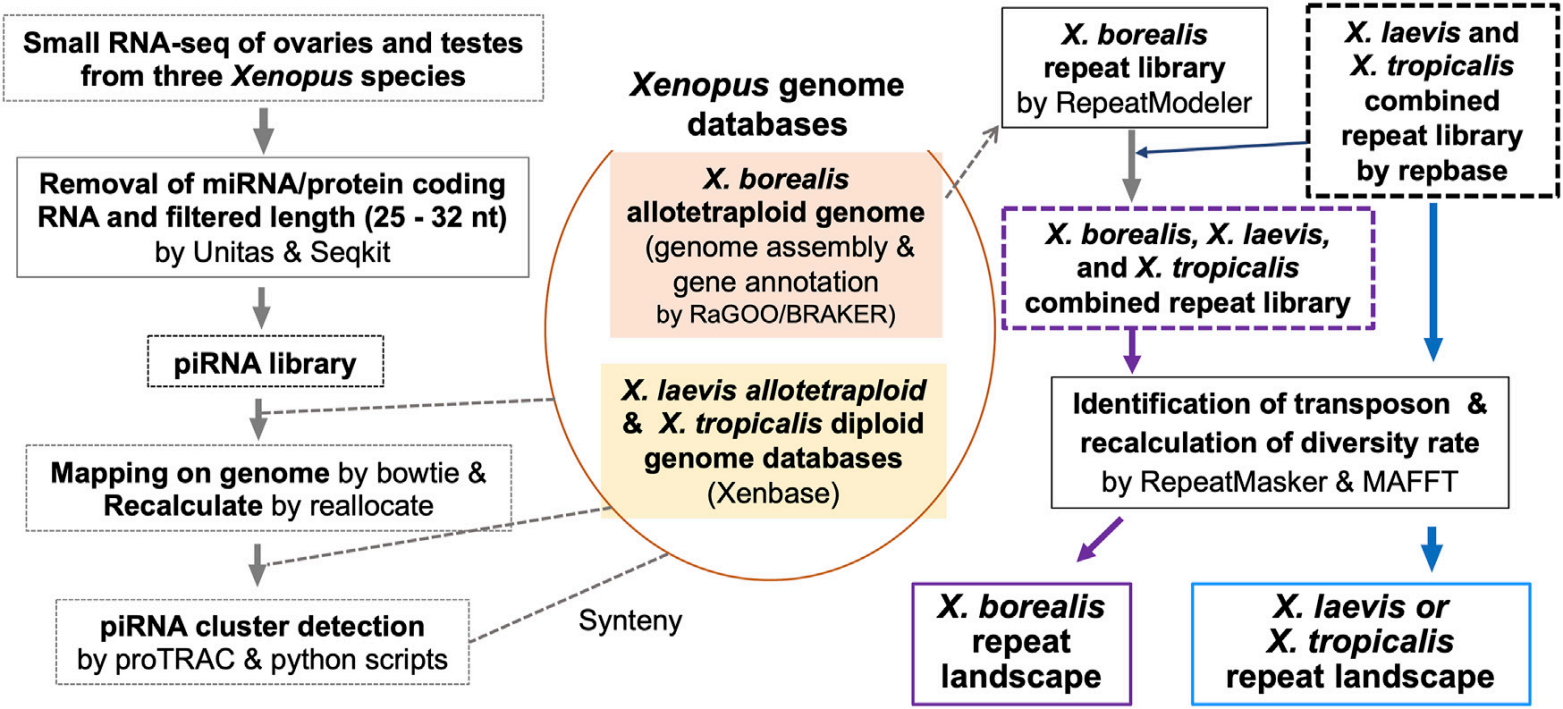
Strategy for genome-wide analysis of piRNAs and repeat sequences including TEs in a diploid and two allotetraploid *Xenopus* frog species.

**FIGURE 2:**
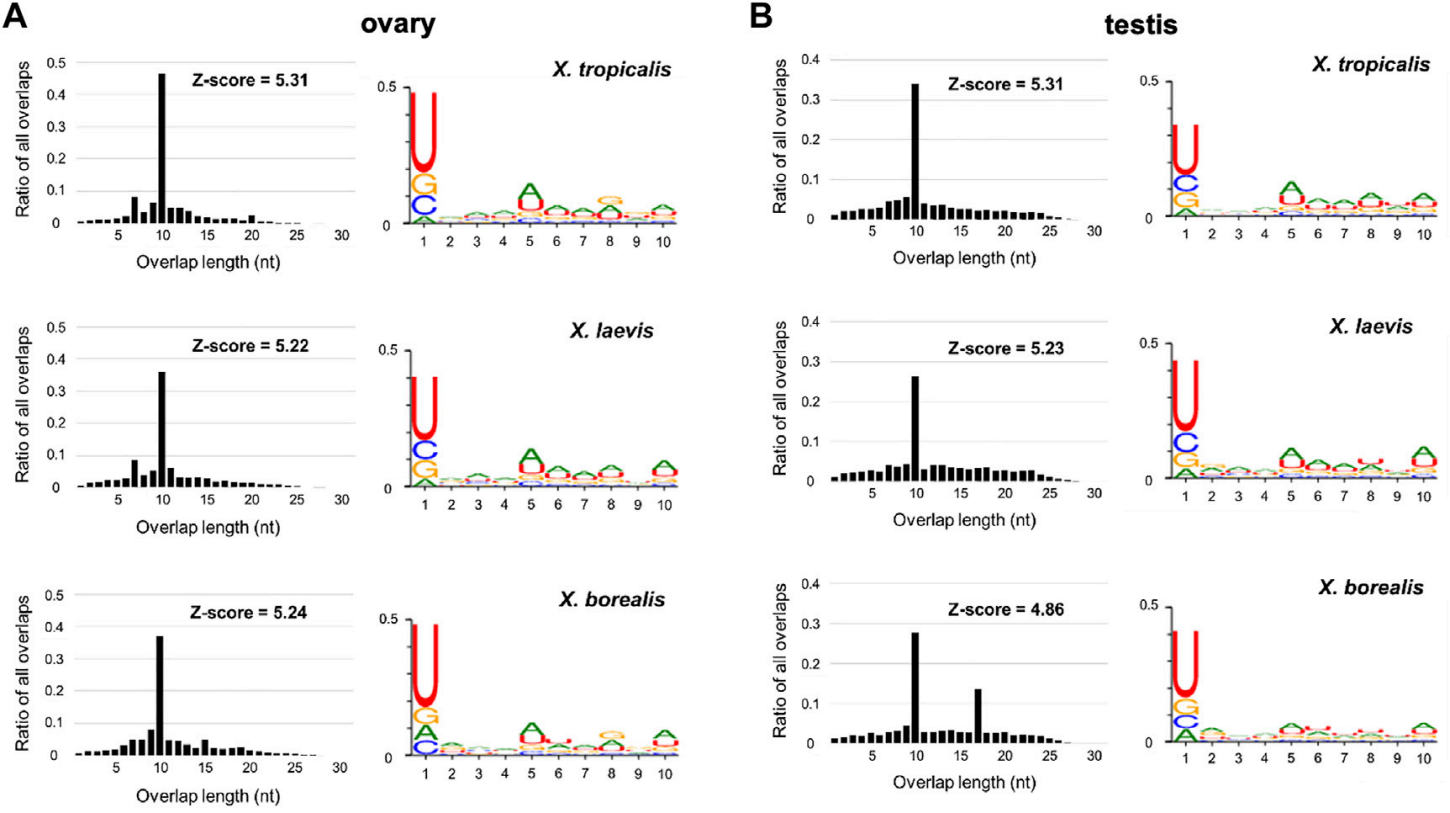
Sequence characterization of ovarian and testicular piRNAs in the three *Xenopus* species, *X. tropicalis*, *X. laevis*, and *X. borealis*. Distributions of 5′-overlap lengths (left) and 1 U/10 A bias (right) on piRNA molecules transcribed in ovaries **(A)** and testes **(B)**. The amounts of piRNAs were normalized by 10^7^/mapped piRNA counts in length distribution. Numbers of sequence types of piRNAs transcribed in testes and ovaries are shown in Venn diagrams **(C)**.

Predicted piRNA molecules were selected from comprehensive sequences of small RNAs from the ovaries and testes of the three *Xenopus* species (Section 2; Figure 1). Almost all the sequences have two characteristics of piRNAs, a high relative amount of 10 nt overlap from 5′ ends and 1 U/10 A biases (Figure 2). Most of the piRNA sequences from each gonad of the three species contained similar numbers of plus and minus strand sequences at every nucleotide length (Supplementary Figure S1), suggesting that they were produced through ping-pong cycles. We also found that there were more than 10^7^ piRNAs in each gonad from the three species, and more than 90% of the piRNAs were distinct between the ovary and testis from each species (Supplementary Figure S2).

### Most piRNA Sequences Are Derived From Retrotransposons, but Also DNA Transposons and Genes in the *Xenopus* Gonads

Most piRNAs are believed to be derived from TEs. We then examined the origin of each piRNA sequence in *X. tropicalis*, *X. laevis*, and *X. borealis*. We first classified the three genomic DNAs into eight groups: DNA transposons, retrotransposons, satellite DNA, simple repeat DNA, exons, introns, others, and unknown sequences in each genome (Section 2). We found that there were similar composition percentages in selfish DNA in all three *Xenopus* genomes: 23–26% of DNA transposons, 8.3–8.8% of retrotransposons, and less than 2% of satellite DNA or simple repeats (Table 1). The percentage of exons (1.9%) and introns (10%) in *X. borealis* were much lower than those (5.0–5.6% and 28–30%, respectively) of the other two species, maybe because “others” in *X. borealis* occupied a higher percentage (50%) from the insufficiency of genome assembly data (Table 1).

**TABLE 1:** Proportion (%) of each DNA or RNA component in genomic DNA or total piRNAs of *X. tropicalis* (*Xt*), *X. laevis* (*Xl*), and *X. borealis* (*Xb*).

	% of genome			Ovary (% of total piRNAs)			Testis (% of total piRNAs)		
	Xt	Xl	Xb	Xt	Xl	Xb	Xt	Xl	Xb
DNA transposon	24	26	23	18	8.7	8.9	8.5	10	11
Retro transposon	8.8	8.3	8.6	40	37	41	23	20	25
Satellite	1.7	0.40	1.8	0.027	0.039	4.4	0.094	0.011	0.34
Simple repeat	0.86	0.68	1.2	0.27	0.41	0.76	0.39	0.66	0.79
Unknown	0.33	0.22	4.0	0.43	0.083	3.4	0.16	0.089	1.8
Exon	5.6	5.0	1.9	6.6	3.4	4.3	14	8.9	7.6
Intron	30	28	10	21	18.4	12	27	28	16
Other	29	32	50	14	32	25	27	32	37

Next, we mapped the obtained piRNA sequences to the classified genomes. We found that all of the DNA transposon-, retrotransposon-, exon-, and intron-derived piRNAs from the ovaries and testes of the three species shared the two characteristics of piRNAs as the unclassified piRNAs did in Figure 2 (Supplementary Figure S3). Interestingly, retrotransposon-derived piRNAs occupied higher percentages in the ovaries (37–41%) than in the testes (20–25%) in total piRNAs in all three species (Table 1). In addition, we found that DNA transposon- and gene (intron and exon)-derived piRNAs were substantially transcribed in both the ovaries and testes of the three *Xenopus* species (Table 1), although piRNAs have been reported to be mostly transcribed from retrotransposons within mammalian genomes (Girard et al., 2006).

### piRNA Clusters Are Not Well Conserved Between the Allotetraploid L and S Subgenomes

Most piRNAs are believed to be transcribed from genomic piRNA clusters (Rosenkranz et al., 2021). We then identified piRNA clusters in the three *Xenopus* genomes from the ovarian and testicular piRNA sequences (Section 2; Figure 1). The number of piRNA clusters was approximately 14, 17, and 13 thousand in the diploid *X. tropicalis,* allotetraploid *X. laevis,* and *X. borealis*, respectively (Supplementary Table S1), indicating larger numbers in *X. tropicalis* in view of genome size. Interestingly, in all three species, the relatively higher density regions of the piRNA clusters were distinct between each ovary and testis, and more than half of the piRNA clusters transcribed specific expression in the ovaries or testes (Supplementary Figure S4; Supplementary Tables S1).

The piRNA clusters are conserved across related species in mammals (Girard et al., 2006; Gebert et al., 2019). To clarify the conservation of piRNA clusters during *Xenopus* diversification, we performed synteny and homology analyses of these clusters not only among the three *Xenopus* species but also between the L and S subgenomes of the two allotetraploid species. In view of chromosomal locations, the piRNA clusters were moderately conserved (approximately 33–59%) in every combination (Supplementary Table S2).

We next examined each proportion of the eight types of DNA, as shown in Table 1, in the ovarian or testicular piRNA clusters from the three *Xenopus* species (Supplementary Figure S5). The proportion of piRNA DNA to retrotransposons in the piRNA clusters was more than two times higher than that of retrotransposon DNA in the genomes of the three species. In contrast, the proportion of piRNAs to DNA transposons in the piRNA clusters was slightly lower than that of DNA transposon-derived DNA in all (sub)genomes. In addition, there were few significant differences in their compositions between the L and S subgenomes of the two allotetraploid species.

### Distinct Landscapes of DNA Transposons and Retrotransposons Between the Diploid and Allotetraploid *Xenopus* Species

**FIGURE 3:**
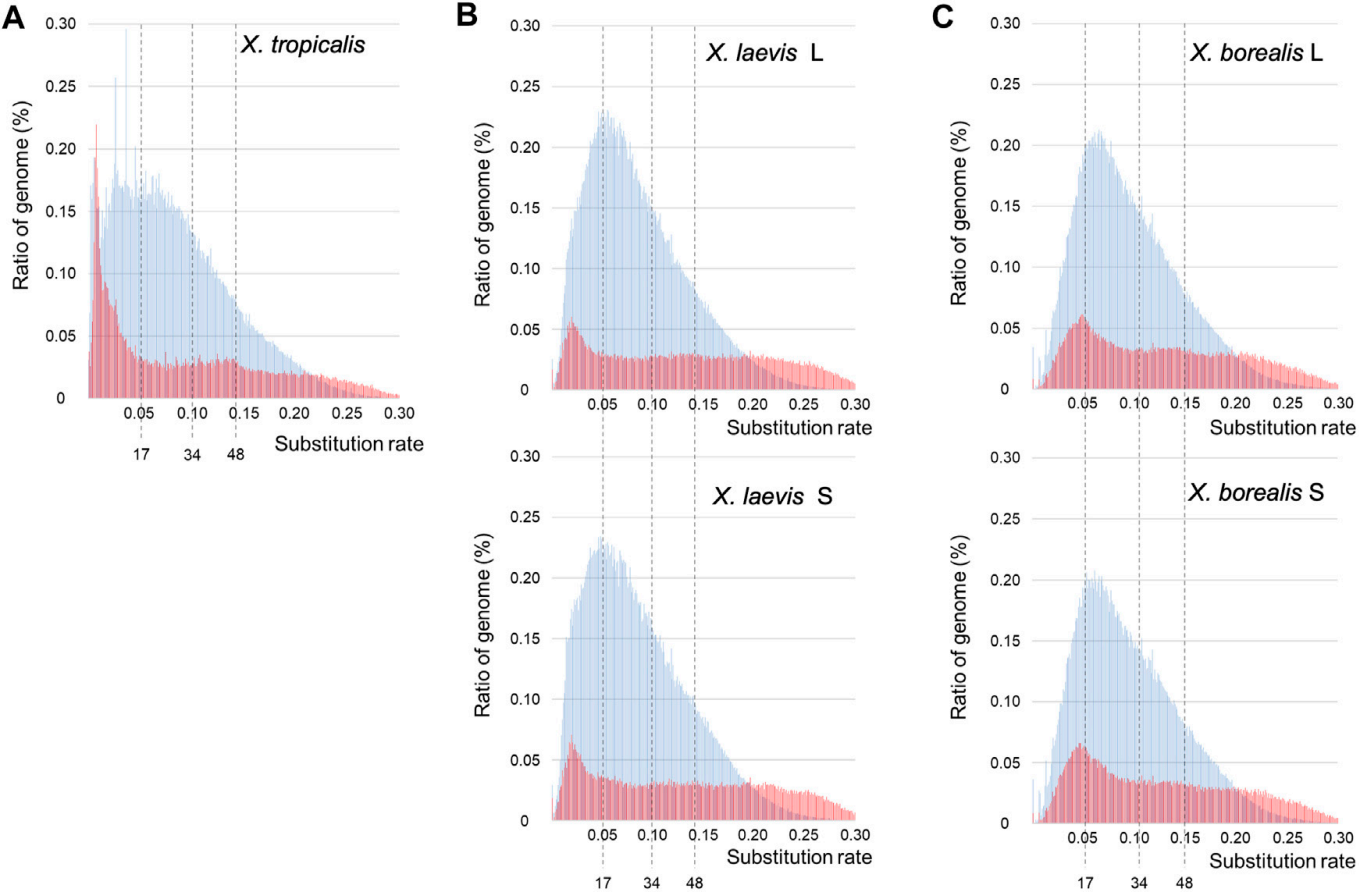
Landscapes of DNA transposons and retrotransposons in the three *Xenopus* species. The substitution ratios of DNA transposons (blue) and retrotransposons (red) in *X. tropicalis* **(A)**, *X. laevis* L and S **(B)**, and *X. borealis* L and S **(C)** are shown on the horizontal axis. Proportion (%) of the transposons to each genome DNA are shown on the vertical axis. The numbers 34 and 17 indicate speciation and hybridization time (mya) of the predicted L and S species, which correspond to the substitution ratios, 0.10 and 0.05, respectively.

The findings of the piRNAs and their clusters in the three *Xenopus* species (Supplementary Figures S2, S4; Supplementary Tables S1, S2) indicate their divergent evolution, suggesting drastic evolution of “transposon-piRNA” system during *Xenopus* species diversity. To clarify the fast and drastic evolution of TEs during species diversification in the *Xenopus* frogs, we examined their nucleotide variations using the genome information of the three species and constructed a TE landscape from their variation frequencies in each genome (Section 2; Figure 1). Most inactive TEs are selectively neutral. Therefore, we adopted 0.01 nucleotide substitution to 3.23–3.33 mya as molecular clock, which was reported in *Xenopus* species by Session et al. (2016). Accordingly, 17 and 34 mya in Figure 3 correspond to the times during the hybridization between the two diploid species with the L- an S-derived genomes and the speciation time to the two ancestral species, respectively. The landscapes of TEs in *the X. tropicalis* diploid genome and L/S subgenomes from allotetraploid *X. laevis* and *X. borealis* are shown in Figure 3. The landscape of the *X. tropicalis* genome was completely different from that of the allotetraploid L/S subgenomes. Both the DNA transposons and retrotransposons appeared to be active until recently in *X. tropicalis* (Figure 3A). In contrast, *X. laevis* and *X. borealis* L and S subgenomes have similar landscapes (Figures 3B,C). Interestingly, the four subgenomes shared large peaks of DNA transposons around 17 mya. These findings indicate that the mixture of the two genomes might be involved in fast and drastic evolution of TEs, especially DNA transposons.

### Activation of a DNA Transposon Superfamily *Kolobok* Around the Interspecific Hybridization

**FIGURE 4:**
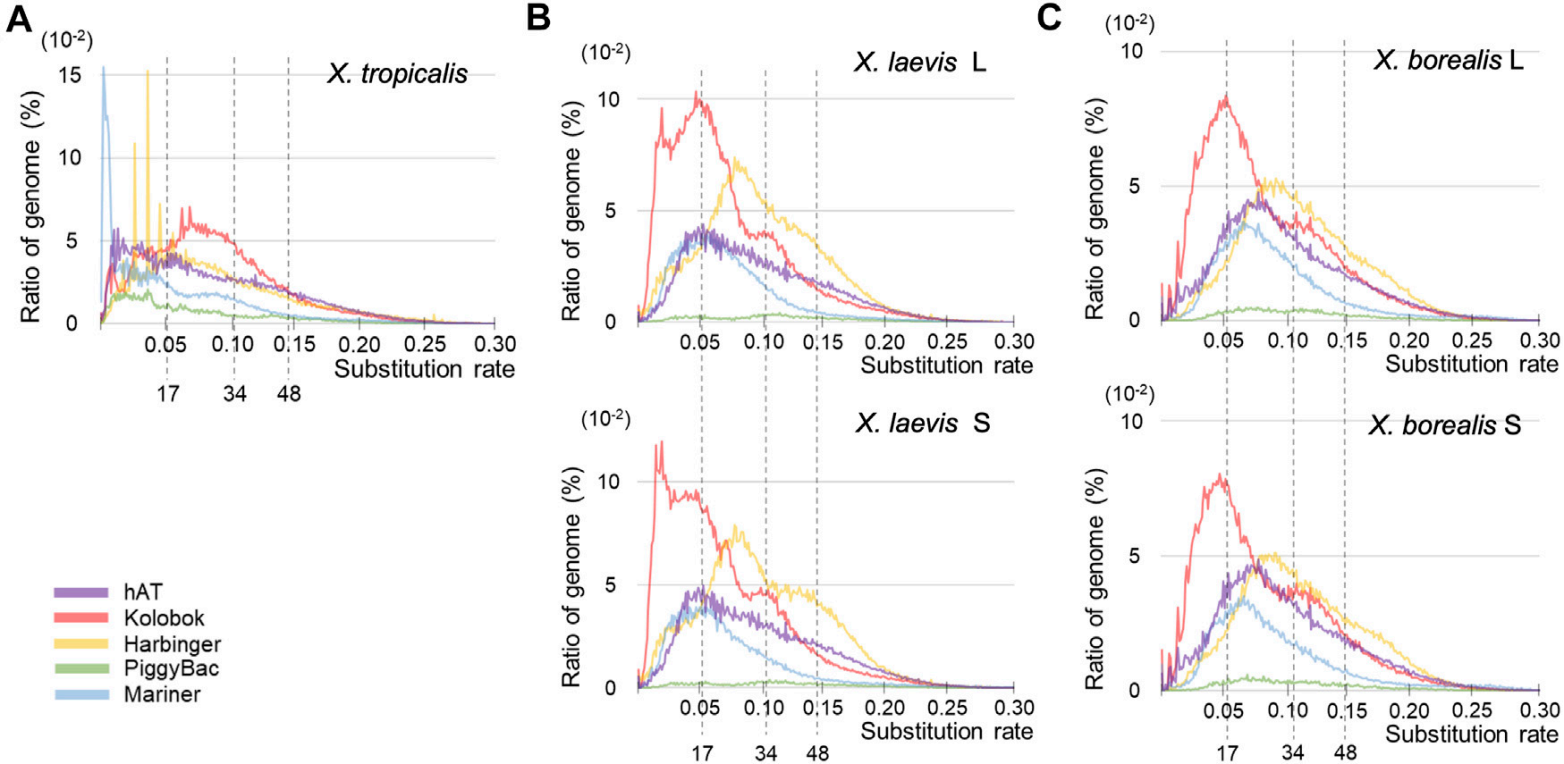
Landscapes of six major superfamilies of DNA transposons in the three *Xenopus* species. The substitution ratio of each superfamily in *X. tropicalis* **(A)**, *X. laevis* L and S **(B)**, and *X. borealis* L and S **(C)** are shown on the horizontal axis. Proportion (%) of the transposons to each genome DNA are shown on the vertical axis.

Since DNA transposons appeared to be activated around the hybridization in the common ancestor of *X. laevis* and *X. borealis* followed by inactivation, we analyzed sequence variations of five major superfamilies (Figure 4). Similar to the results in Figure 3, the landscape from *X. tropicalis* was unique (Figure 4A), whereas those of the five superfamilies were similar between the two L and S subgenomes of *X. laevis* and *X. borealis* (Figures 4B,C). Notably, the *Kolobok* superfamily showed activity peaks at approximately 17 mya in both *X. laevis* L/S and *X. boralis* L/S subgenomes. The *Kolobok* superfamily also showed another peak around 7 mya in the L and S subgenomes in *X. laevis*, but not in *X. borealis.* In contrast, activity peaks around 17 mya were observed in *hAT* and *Mariner* superfamilies from *X. laevis* L/S, but not *in X. borealis* L/S subgenomes. These findings suggest that the high activity of some transposon superfamilies including *Kolobok* could be involved in the hybridization event with the mingled state of two distinct genomes.

## Discussion

In this study, we obtained ovarian and testicular piRNA sequences from the three *Xenopus* species and analyzed them (Figure 2; Supplementary Figures S1–S5; Supplementary Tables S1, S2). All of the samples have two characteristics of piRNAs, 1U/10A bias and the peak of 10 nucleotide overlap from 5′ (Figure 2). Curiously, we observed not only 10, but also 17 significant overlaps in the *X. borealis* testicular piRNAs (Figure 2). However, we could not understand the reason. Less than 10% of the total types of piRNAs were common between the ovary and testis in each species (Supplementary Figure S2). We do not know whether the differences between them was caused mainly by female and male gonads or each individual. In several actinopterygian fish, ovary and testis-specific piRNAs have been reported (Wang et al., 2016; Yuan et al., 2019). Faunes et al. (2012) reported that in *X. tropicalis* a large number of transposon-derived piRNAs are present in gastrula embryos, most of which are shared with oocytes. Furthermore, satellite repeat-derived piRNAs regulate the embryonic development of an insect (Halbach et al., 2020). It will be interesting to study whether some oocyte-derived piRNAs control embryogenesis, such as repression of selfish DNA and RNA.

By mapping the piRNA sequences to their corresponding genomes, we specified the piRNA clusters transcribed in female and male gonads in the three *Xenopus* species, indicating that almost all the piRNA clusters were not well conserved among the three species or between the allotetraploid-derived L and S subgenomes (Table 1; Supplementary Tables S1, S2; Supplementary Figure S4). Therefore, piRNA clusters might have evolved drastically during species diversity in *Xenopus* frogs. Toombs et al. (2017) reported that *Xenopus* piRNAs were identified as PIWI-interacting RNAs from early stage oocytes and that PIWI could promote the turnover of TEs and other RNAs and regulate mRNA localization and translation in germ cells. Because the positions of the piRNA clusters were not shown in the report, we could not directly compare their data with those obtained in this study. Then we compared the piRNA clusters between the updated database of piRNA cluster (www.smallrnagroup.uni-mainz.de/piRNAclusterDB/) and this study. More than half of piRNA clusters (391/566 and 302/480) in the updated database from *X. laevis* and *X. tropicalis*, respectively, were merged to those identified in this study. The differences might be supported in part by the fact that the frogs in both the species were not derived from the inbred lines. Gebert et al. (2019) reported that some piRNA clusters were well conserved during primate evolution. This difference might be related to the higher percentage of DNA transposons in *Xenopus* frog genomes, as shown in Table 1, than in primate genomes.

Although piRNAs and PIWI are well known to silence retrotransposons by heterochromatin formation of their genes or cleavage of their transcripts (Russell and LaMarre, 2021), it remains unclear whether some piRNA molecules could regulate DNA transposons and how they would do so. In many mammals, the majority of TEs originate from retrotransposons (Vandewege et al., 2016). In contrast, DNA transposons are dominant among TEs in amphibian genomes (Hellsten et al., 2010; Session et al., 2016; Table 1). Here, we found that DNA transposon-derived piRNAs accounted for 5–12% of total piRNAs in female and male gonads from the three *Xenopus* species (Table 1). It is interesting to study whether DNA transposon-derived piRNAs could regulate DNA transposons during gametogenesis and embryogenesis and how they would do so.

Interspecific hybridization has been reported to cause the proliferation of transposons in some bilaterian animal hybrids such as fruit flies in the *Drosophila* genus and teleost fish sculpins in the *Cottus* genus (Khurana et al., 2011; Dennenmoser et al., 2019; Kotov et al., 2019). In the TE landscapes, large peaks of total DNA transposons were observed around the hybridization time (17 mya) in the two hybrid-derived species *X. laevis* and *X. borealis*, but not in the diploid species *X. tropicalis* (Figure 3). Moreover, we observed that several DNA transposon superfamilies including *Kolobok* were activated around the hybridization time (Figure 4). In general, the genomes of two closely related species had few differences in gene sequences, but contain distinct TEs and piRNA genes, including piRNA clusters. Here, we confirmed the differences in the piRNA clusters in the three *Xenopus* genomes (Supplementary Table S2). The interspecific hybridization, which was accompanied with the mixture of the two distinct *Xenopus* genomes, might induce activation of DNA transposons such as the *Kolobok* superfamily because of the two distinct “transposon-piRNA” systems. The activities of the two superfamilies were repressed by their corresponding piRNAs that newly emerged piRNA genes should encode. We speculate that the asymmetric evolution between the L and S subgenomes in the allotetraploid *Xenopus* species has been caused, in part, by activation of the DNA transposon and repression of their corresponding piRNAs around and after hybridization.

## Data Availability

The datasets presented in this study can be found in online repositories. The names of the repository/repositories and accession number(s) can be found below: DNA data bank (DDBJ), PRJDB12462.

## References

[B1] Alonge M. Soyk S. Ramakrishnan S. Wang X. Goodwin S. Sedlazeck F. J. (2019). RaGOO: Fast and Accurate Reference-Guided Scaffolding of Draft Genomes. Genome Biol. 20, 224. 10.1186/s13059-019-1829-6 31661016 PMC6816165

[B2] Belancio V. P. Hedges D. J. Deininger P. (2008). Mammalian Non-LTR Retrotransposons: for Better or Worse, in Sickness and in Health. Genome Res. 18, 343–358. 10.1101/gr.5558208 18256243

[B3] Caballero M. Wegrzyn J. (2019). gFACs: Gene Filtering, Analysis, and Conversion to Unify Genome Annotations across Alignment and Gene Prediction Frameworks. Genomics, Proteomics & Bioinformatics 17, 305–310. 10.1016/j.gpb.2019.04.002 PMC6818179 31437583

[B4] Dennenmoser S. Sedlazeck F. J. Schatz M. C. Altmüller J. Zytnicki M. Nolte A. W. (2019). Genome‐wide Patterns of Transposon Proliferation in an Evolutionary Young Hybrid Fish. Mol. Ecol. 28, 1491–1505. 10.1111/mec.14969 30520198

[B5] Faunes F. Almonacid L. I. Melo F. Larrain J. (2012). Characterization of Small RNAs inXenopus Tropicalisgastrulae. Genesis 50, 260–270. 10.1002/dvg.22012 22253037

[B6] Gebert D. Hewel C. Rosenkranz D. (2017). Unitas: the Universal Tool for Annotation of Small RNAs. BMC Genomics 18, 644. 10.1186/s12864-017-4031-9 28830358 PMC5567656

[B7] Gebert D. Zischler H. Rosenkranz D. (2019). Primate piRNA Cluster Evolution Suggests Limited Relevance of Pseudogenes in piRNA-Mediated Gene Regulation. Genome Biol. Evol. 11, 1088–1104. 10.1093/gbe/evz060 30888404 PMC6461890

[B8] Girard A. Sachidanandam R. Hannon G. J. Carmell M. A. (2006). A Germline-specific Class of Small RNAs Binds Mammalian Piwi Proteins. Nature 442, 199–202. 10.1038/nature04917 16751776

[B9] Halbach R. Miesen P. Joosten J. Taşköprü E. Rondeel I. Pennings B. (2020). A Satellite Repeat-Derived piRNA Controls Embryonic Development of Aedes. Nature 580, 274–277. 10.1038/s41586-020-2159-2 32269344 PMC7145458

[B10] Hellsten U. Harland R. M. Gilchrist M. J. Hendrix D. Jurka J. Kapitonov V. (2010). The Genome of the Western Clawed Frog Xenopus Tropicalis. Science 328, 633–636. 10.1126/science.1183670 20431018 PMC2994648

[B11] Hoff K. J. Lomsadze A. Borodovsky M. Stanke M. (2019). Whole-Genome Annotation with BRAKER. Methods Mol. Biol. 1962, 65–95. 10.1007/978-1-4939-9173-0_5 31020555 PMC6635606

[B12] Holland P. W. H. Garcia-Fernàndez J. Williams N. A. Sidow A. (1994). Gene Duplications and the Origins of Vertebrate Development. Dev. Suppl. 1994, 125–133. 10.1242/dev.1994.supplement.125 7579513

[B13] Khurana J. S. Wang J. Xu J. Koppetsch B. S. Thomson T. C. Nowosielska A. (2011). Adaptation to P Element Transposon Invasion in *Drosophila melanogaster* . Cell 147, 1551–1563. 10.1016/j.cell.2011.11.042 22196730 PMC3246748

[B14] Klattenhoff C. Bratu D. P. McGinnis-Schultz N. Koppetsch B. S. Cook H. A. Theurkauf W. E. (2007). Drosophila rasiRNA Pathway Mutations Disrupt Embryonic axis Specification through Activation of an ATR/Chk2 DNA Damage Response. Dev. Cel 12, 45–55. 10.1016/j.devcel.2006.12.001 17199040

[B15] Kotov A. A. Adashev V. E. Godneeva B. K. Ninova M. Shatskikh A. S. Bazylev S. S. (2019). piRNA Silencing Contributes to Interspecies Hybrid Sterility and Reproductive Isolation in *Drosophila melanogaster* . Nucleic Acids Res. 47, 4255–4271. 10.1093/nar/gkz130 30788506 PMC6486647

[B16] Miura I. Vershinin V. Vershinina S. Lebedinskii A. Trofimov A. Sitnikov I. (2021). Hybridogenesis in the Water Frogs from Western Russian Territory: Intrapopulation Variation in Genome Elimination. Genes 12, 244. 10.3390/genes12020244 33567735 PMC7914630

[B17] Ohno S. (1970). Evolution by Gene Duplication. Berlin: Springer.

[B18] Pertea M. Pertea G. M. Antonescu C. M. Chang T.-C. Mendell J. T. Salzberg S. L. (2015). StringTie Enables Improved Reconstruction of a Transcriptome from RNA-Seq Reads. Nat. Biotechnol. 33, 290–295. 10.1038/nbt.3122 25690850 PMC4643835

[B19] Quinlan A. R. Hall I. M. (2010). BEDTools: a Flexible Suite of Utilities for Comparing Genomic Features. Bioinformatics 26, 841–842. 10.1093/bioinformatics/btq033 20110278 PMC2832824

[B20] Rosenkranz D. Zischler H. Gebert D. (2021). piRNAclusterDB 2.0: Update and Expansion of the piRNA Cluster Database. Nucleic Acids Res. 50, D259–D264. 10.1093/nar/gkab622 PMC8728273 34302483

[B21] Rosenkranz D. Zischler H. (2012). proTRAC - a Software for Probabilistic piRNA Cluster Detection, Visualization and Analysis. BMC Bioinformatics 13, 5. 10.1186/1471-2105-13-5 22233380 PMC3293768

[B22] Russell S. J. LaMarre J. (2018). Transposons and the PIWI Pathway - Genome Defence in Gametes and Embryos. Reproduction 156, R111–R124. 10.1530/REP-18-0218 30304932

[B23] Session A. M. Uno Y. Kwon T. Chapman J. A. Toyoda A. Takahashi S. (2016). Genome Evolution in the Allotetraploid Frog Xenopus laevis. Nature 538, 336–343. 10.1038/nature19840 27762356 PMC5313049

[B24] Seto A. G. Kingston R. E. Lau N. C. (2007). The Coming of Age for Piwi Proteins. Mol. Cel 26, 603–609. 10.1016/j.molcel.2007.05.021 17560367

[B25] Simakov O. Marlétaz F. Yue J.-X. O’Connell B. Jenkins J. Brandt A. (2020). Deeply Conserved Synteny Resolves Early Events in Vertebrate Evolution. Nat. Ecol. Evol. 4, 820–830. 10.1038/s41559-020-1156-z 32313176 PMC7269912

[B26] Siomi M. C. Sato K. Pezic D. Aravin A. A. (2011). PIWI-interacting Small RNAs: the Vanguard of Genome Defence. Nat. Rev. Mol. Cel Biol. 12, 246–258. 10.1038/nrm3089 21427766

[B27] Spring J. (1997). Vertebrate Evolution by Interspecific Hybridisation - Are We Polyploid? FEBS Lett. 400, 2–8. 10.1016/s0014-5793(96)01351-8 9000502

[B28] Toombs J. A. Sytnikova Y. A. Chirn G.-w. Ang I. Lau N. C. Blower M. D. (2017). Xenopus Piwi Proteins Interact with a Broad Proportion of the Oocyte Transcriptome. RNA 23, 504–520. 10.1261/rna.058859.116 28031481 PMC5340914

[B29] Vandewege M. W. Platt R. N. Ray D. A. Hoffmann F. G. (2016). Transposable Element Targeting by piRNAs in Laurasiatherians with Distinct Transposable Element Histories. Genome Biol. Evol. 8, 1327–1337. 10.1093/gbe/evw078 27060702 PMC4898795

[B30] Wang C. L. Wang Z. P. Wang J. Q. Li M. Y. Chen X. W. (2016). Identification of Candidate piRNAs in the Gonads of Paralichthys olivaceus (Japanese Flounder). Zool Res. 37, 301–306. 10.13918/j.issn.2095-8137.2016.5.301 27686790 PMC5071344

[B31] Yuan L. Li L. Zhang X. Jiang H. Chen J. (2019). Identification and Differential Expression of piRNAs in the Gonads of Amur sturgeon (Acipenser Schrenckii). PeerJ 7, e6709. 10.7717/peerj.6709 31106045 PMC6499119

