# Supplementary material for: Activation of DNA Transposons and Evolution of piRNA Genes Through Interspecific Hybridization in Xenopus Frogs
Source: Front Genet. 2022 Jan 31;13:766424. doi: 10.3389/fgene.2022.766424 (PMC8841583; doi:10.3389/fgene.2022.766424)
Supplement: Supplementary file 1 [file DataSheet1.PDF]

Supplementary Information

Supplementary figures

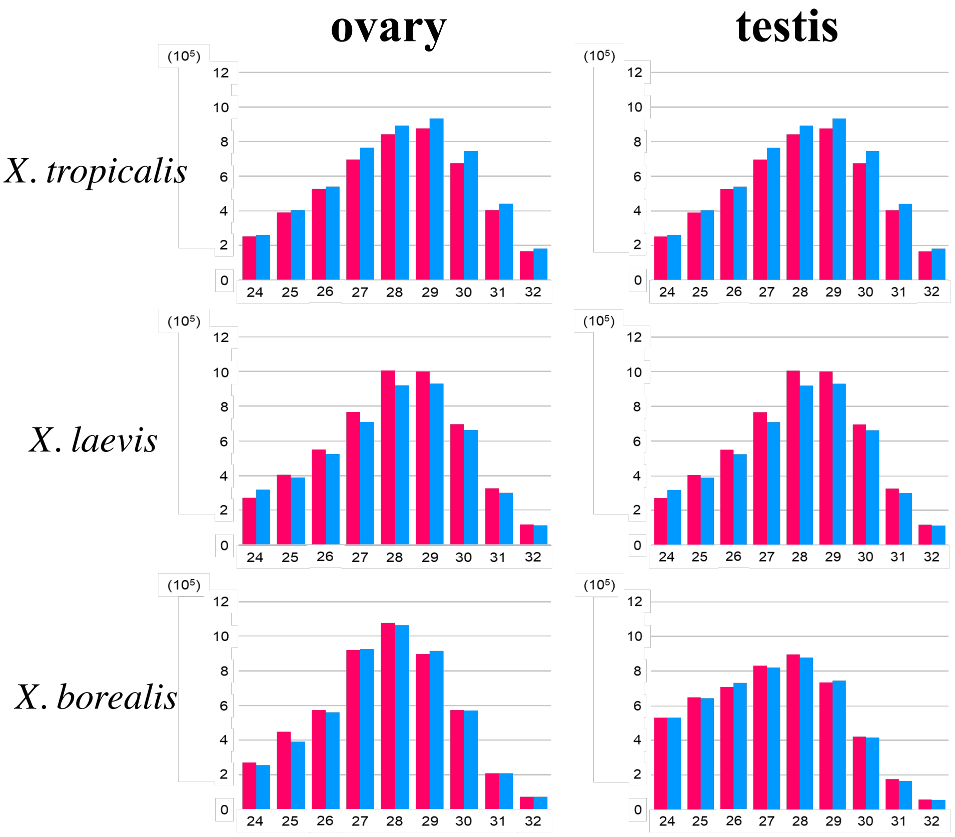

Fig. S1. Distributions of lengths of plus (red) and minus (blue) stranded piRNA molecules transcribed in ovaries and testes.

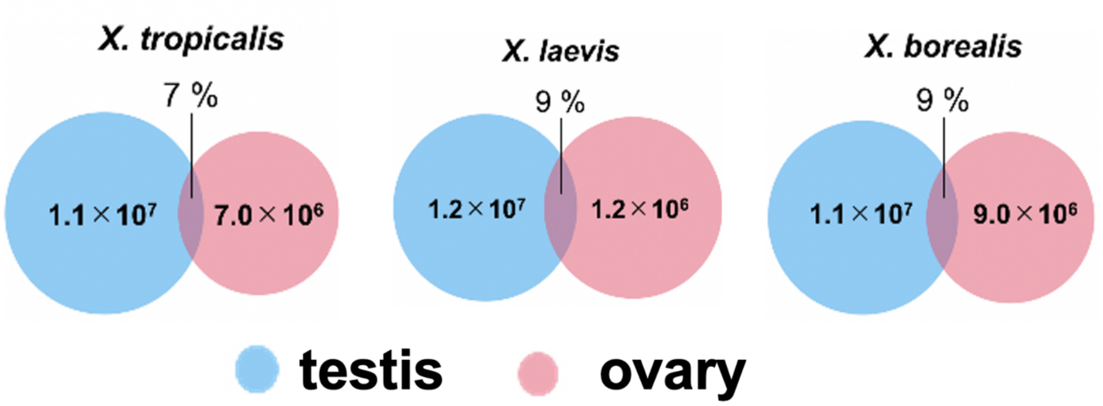

Fig. S2. Numbers of testicular and ovarian piRNAs from three *Xenopus* frog species in venn diagrams. The intersection of the diagram corresponds to the identical sequences between the two.

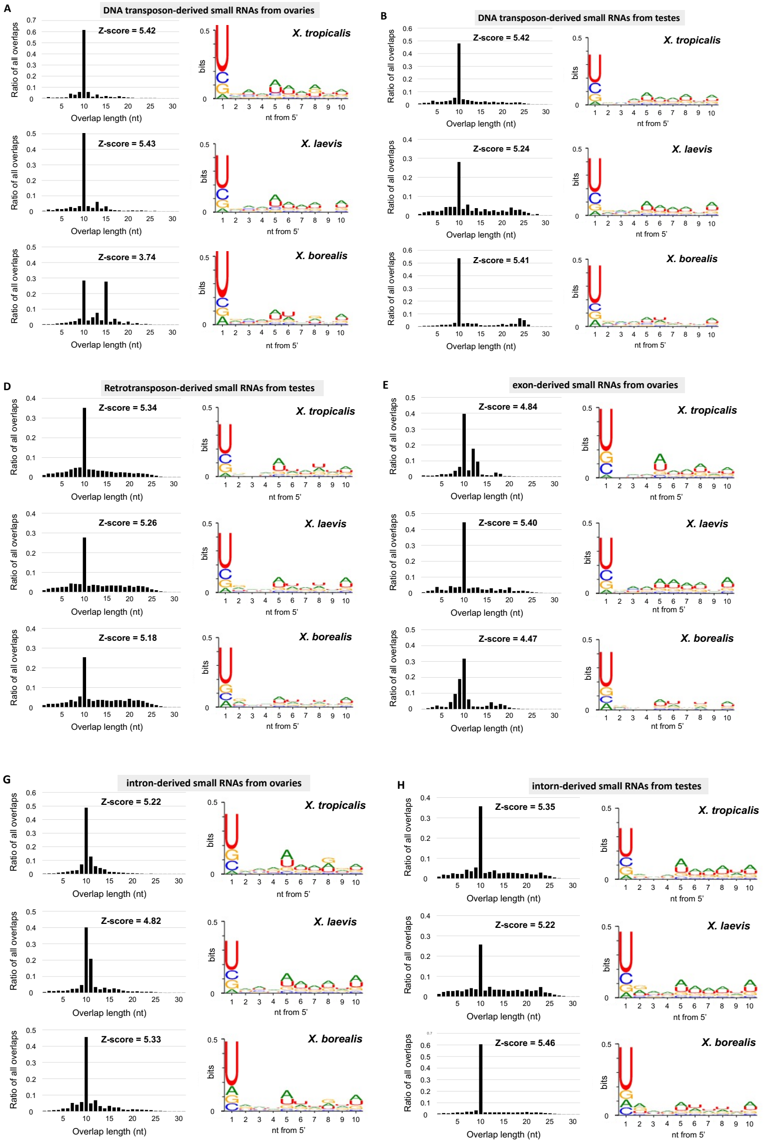

Fig. S3. Distributions of 5'-overlap lengths and 1U/10A bias on four types of piRNA molecules from ovaries and testes in three *Xenopus* species. Four types include DNA transposon-derived piRNAs (A and B), retrotransposon-derived piRNAs (C and D), exon-derived piRNAs (E and F), and intron-derived piRNAs (G and H). A, C, E, and G, or B, D, F, and H indicate ovarian or testicular piRNAs, respectively.

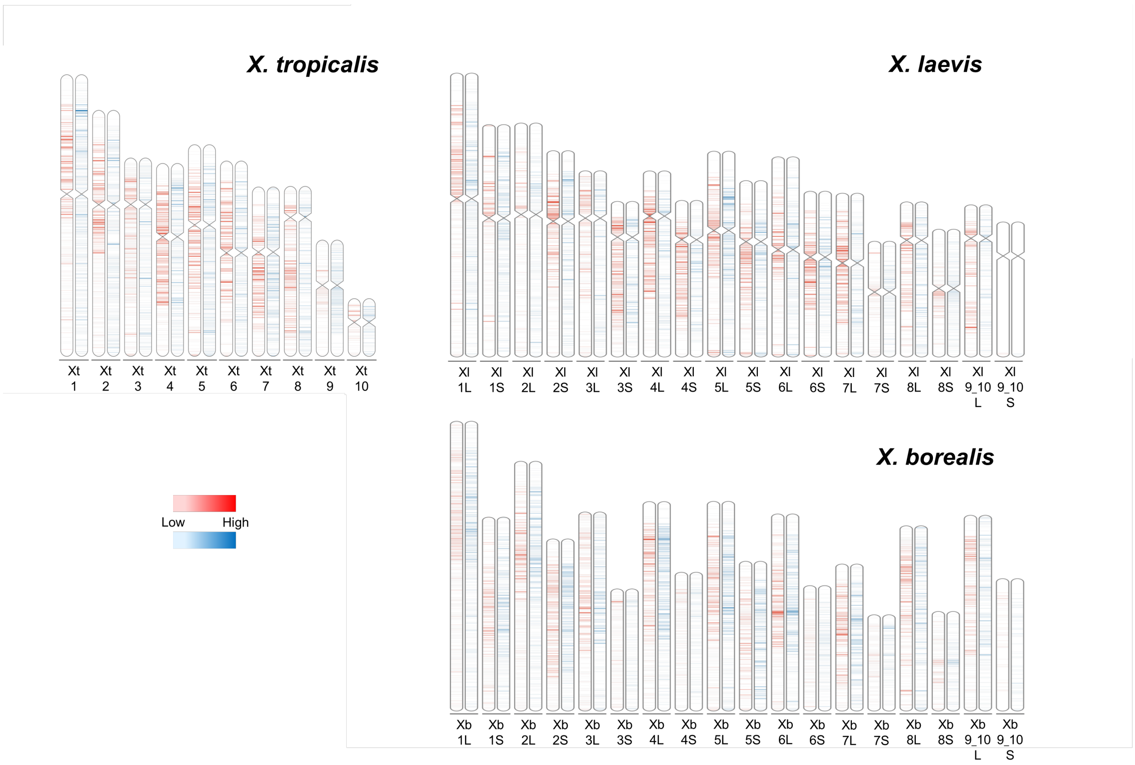

Fig. S4. Positions of piRNA clusters transcribed in ovaries and testes on each chromosome of *X. tropicalis* (Xt), *X. laevis* (Xl) and *X. borealis* (Xb). Red and blue lines indicate the positions of the clusters from ovaries and testes, respectively, on each chromosome.

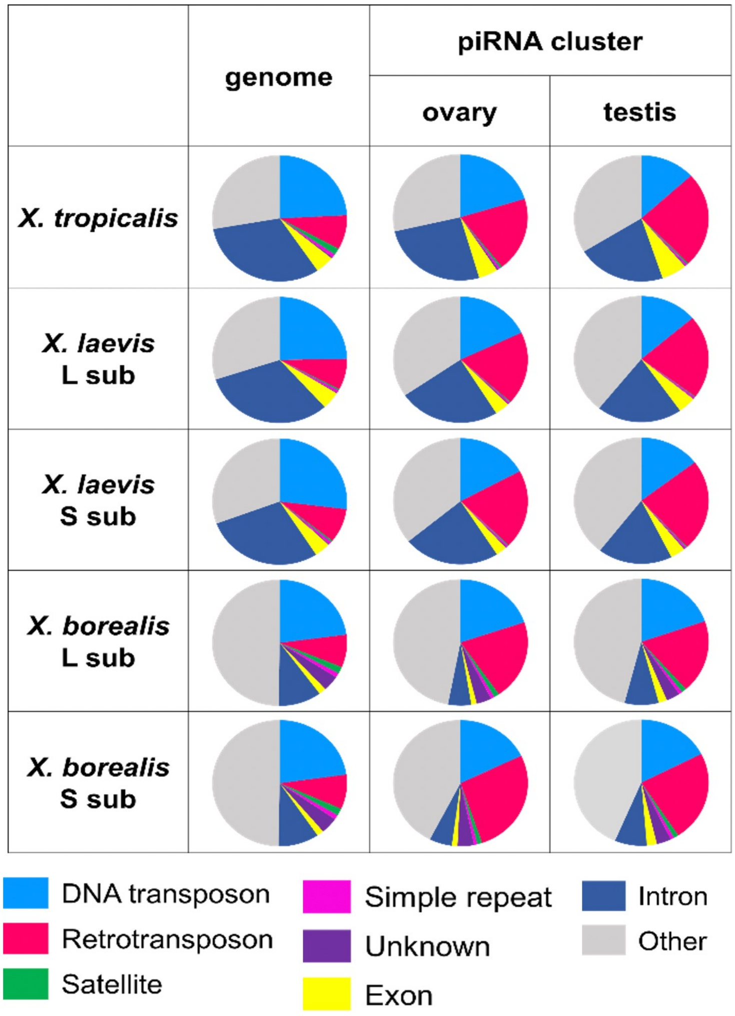

Fig. S5. Percentage of DNA components to (sub)genomes and piRNA clusters from ovaries and testes in the three *Xenopus* species.

Supplementary tables

Table S1. The numbers of piRNA clusters transcribed in ovaries and testes in *Xenopus* genomes

	ovary	testis	common (%)	total
Xt	5065	10845	921 (27)	14068
Xl L	4869	6330	638 (23)	9923
Xl S	3952	3674	420 (22)	6786
Xb L	5314	4847	904 (36)	8353
Xb S	2994	2690	490 (35)	4704

Xt, Xl, and Xb indicate *X. tropicalis*, *X. laevis* and *X. borealis*, respectively.

Table S2. Proportion (%) of conserved piRNA clusters in chromosomal locations between the two (sub)genomes from *X. tropicalis* (Xt) genome, *X. laevis* (Xl) and *X. borealis* (Xb) L/S subgenomes.

	Xt (%)	Xl L (%)	Xl S (%)	Xb L (%)	Xb S (%)	piC (No.)
Xt		46.9	34.0	49.1	33.4	7302
Xl L			43.0	59.3	37.5	5259
Xl S				54.2	53.4	3858
Xb L					41.6	6246
Xb S						3374

Xt, Xl, and Xb indicate *X. tropicalis*, *X. laevis* and *X. borealis*, respectively.
